# Focused Ultrasound Stimulation of Microbubbles in Combination With Radiotherapy for Acute Damage of Breast Cancer Xenograft Model

**DOI:** 10.1177/15330338221132925

**Published:** 2022-11-22

**Authors:** Deepa Sharma, Farah Hussein, Niki Law, Golnaz Farhat, Christine Tarapacki, Lakshmanan Sannachi, Anoja Giles, Gregory J. Czarnota

**Affiliations:** 1 Sunnybrook Health Sciences Centre, Toronto, ON, Canada; 2 University of Toronto, Toronto, ON, Canada

**Keywords:** breast cancer xenografts, cell death, focused ultrasound, radiation therapy, ultrasound-stimulated microbubbles

## Abstract

**Objective:** Several studies have focused on the use of ultrasound-stimulated microbubbles (USMB) to induce vascular damage in order to enhance tumor response to radiation. **Methods:** In this study, power Doppler imaging was used along with immunohistochemistry to investigate the effects of combining radiation therapy (XRT) and USMB using an ultrasound-guided focused ultrasound (FUS) therapy system in a breast cancer xenograft model. Specifically, MDA-MB-231 breast cancer xenograft tumors were induced in severe combined immuno-deficient female mice. The mice were treated with FUS alone, ultrasound and microbubbles (FUS  +  MB) alone, 8 Gy XRT alone, or a combined treatment consisting of ultrasound, microbubbles, and XRT (FUS  +  MB  +  XRT). Power Doppler imaging was conducted before and 24 h after treatment, at which time mice were sacrificed and tumors assessed histologically. The immunohistochemical analysis included terminal deoxynucleotidyl transferase dUTP nick end labeling, hematoxylin and eosin, cluster of differentiation-31 (CD31), Ki-67, carbonic anhydrase (CA-9), and ceramide labeling. **Results:** Tumors receiving treatment of FUS  +  MB combined with XRT demonstrated significant increase in cell death (p  =  0.0006) compared to control group. Furthermore, CD31 and Power Doppler analysis revealed reduced tumor vascularization with combined treatment indicating (*P* < .0001) and (*P*  =  .0001), respectively compared to the control group. Additionally, lesser number of proliferating cells with enhanced tumor hypoxia, and ceramide content were also reported in group receiving a treatment of FUS  +  MB  +  XRT. **Conclusion:** The study results demonstrate that the combination of USMB with XRT enhances treatment outcomes.

## Introduction

Tumor blood vessels play an important role in providing rapidly dividing tumor cells with oxygen and nutrients.^1^ Hence, damage to tumor vasculature can greatly affect tumor growth.^1–3^ Several studies have investigated the use of ultrasound-stimulated microbubbles (USMB) to induced vascular damage which can directly impact tumor growth or sensitize tumor cells to certain cancer treatment modalities such as chemotherapy and radiation therapy (XRT).^4–8^

Ultrasound imaging often uses gas-filled microbubbles as a contrast agent due to their high echogenicity.^9^ When exposed to ultrasound, microbubbles oscillate in response to the mechanical pressure exerted on them, this process is known as acoustic cavitation. There are 2 types of acoustic cavitation: stable and inertial cavitation.^10^ Stable cavitation occurs at low ultrasound pressures and can be linear or nonlinear depending on ultrasound frequency and pressure amplitude, while inertial cavitation occurs at higher pressures and results in microbubble implosion.^10^ The cavitation of the microbubble can induce shear stress, affecting the surrounding tissue. This has been suggested to have potential therapeutic applications both in vitro and in vivo.^11^

The shear stress induced by USMB within the tumor microvasculature can damage endothelial cells lining the blood vessels. These effects may lead to increased vascular permeability, decreased vascular integrity, and vasoconstriction, subsequently causing vascular shutdown.^12^ In addition, exposure to USMB can result in a decrease in cell viability and an increase in endothelial cell membrane permeability through a process known as sonoporation.^13^ These effects were found to be dependent on treatment parameters such as ultrasound pressure, frequency, exposure time, and microbubble concentration.^14^ The bioeffects of USMB open the door to a range of potential therapeutic applications including targeted drug and gene delivery into cancer cells,^15,16^ induction of vascular damage or vasoconstriction to starve tumor cells, and the sensitization of tumors to anticancer treatments such as chemotherapy and XRT.^17,18^

Recent studies on the radiosensitizing effects of USMB have suggested that the disruption of microvascular endothelial cells results in the activation of cell-death signaling pathways.^5^ The membrane-perturbation caused by USMB can lead to an increase in acid sphingomyelinase (ASMase) activity in endothelial cells, which results in ceramide accumulation and leads to increased cell death through apoptosis. This increase in ASMase-mediated ceramide production is believed to increase the sensitivity of tumors to XRT.^4,5,20^

In the study here, power Doppler imaging was used in combination with immunohistochemical analysis to investigate the effects of combining XRT and USMB using an ultrasound-imaging guided focused ultrasound (FUS) therapy system in a breast cancer xenograft model. The motivation for this work is to build on previous studies suggesting that the stimulation of microbubbles within tumor microvasculature can induce endothelial cell damage that enhances the effects of XRT.^18^ Image-guided ultrasound therapy has previously been used to enhance the Spatio-temporal control of ultrasound therapy.^21^ The treatment system used here improves spatial specificity by using a FUS transducer that allows for concentrating ultrasound energy in a small focal area and delivering a well-characterized ultrasound therapy beam that is precisely focused at a treatment target with the guidance of a low-frequency ultrasound imaging transducer.

The main hypothesis guiding this study is that the local stimulation of microbubbles within the tumor microvasculature using FUS can enhance the effects of XRT in a breast cancer model. Tumor response assessed at 24 h following treatment demonstrated that the combination of FUS  +  MB with XRT improved the outcome of treatment by reducing tumor vascularization, blood flow, tumor oxygenation, and tumor cell proliferation. Furthermore, increased ceramide labeling and cell death levels were also observed in the combined FUS  +  MB  +  XRT treated group.

## Materials and Methods

The reporting of this study confirms to ARRIVE 2.0 guidelines.^22^ All experimental procedures were conducted in compliance with protocols approved by the Sunnybrook Research Institute Institutional Animal Care and Use Committee (SRI ACC, protocol 447).

### Cell Culture

Human breast adenocarcinoma cells (MDA-MB-231, ATCC, MD, USA) were cultured in tissue culture flasks at 5% CO_2_ and 37 °C in RPMI-1640 medium supplemented with 10% fetal bovine serum and 1% penicillin/streptomycin antibiotics. The cells were harvested by trypsinization using 0.05% trypsin–EDTA (Gibco, Thermo Fisher Scientific) and suspended in Ca^+^/Mg^+^ phosphate buffered saline at a concentration of 5  ×  10^4^ cells/μL.

### Animal Model

Adequate care of the animals was taken following guidelines.^23^ A total number of 25 animals with 5 mice were used per treatment condition. Four-week to 6-week-old female severe combined immunodeficiency mice (Charles River Canada, Saint-Constant, QC, Canada) received an injection of 100 μL of the MDA-MB-231 cell suspension in the right hind leg using a 27-gauge needle. Tumors were allowed to grow and reach an approximate diameter of 7-9 mm with a maximum diameter of 10 mm. Oxygen ventilated isoflurane (2%) was used to anesthetize mice during tail vein cannulation with 25-gauge catheters for microbubble injection. The animals were then injected subcutaneously with 100 µL of a ketamine and xylazine mixture (150 mg/kg ketamine mixed with 10 mg/kg xylazine in saline) prior to ultrasound imaging and treatment. Post-treatment imaging was performed at 24 h. The animals were subsequently sacrificed and tumors were excised for histology and immunohistochemistry. Animals received either no treatment or one of the following treatments: focused ultrasound (FUS) only, radiation (XRT) only, ultrasound and microbubbles (FUS  +  MB), or a combination of ultrasound, microbubbles, and radiation (FUS  +  MB  +  XRT).

Throughout the experiments, mice were visually monitored. To maintain regular body temperature and limit vasoconstriction due to hypothermia during treatment, animals were placed under heat lamps or kept over warmed pads. Oxygen was administered if irregular respiratory rates were noticed in animals.

### Microbubble Preparation

Definity microbubbles (Lantheus Medical Imaging, Billerica, MA, USA) were used in this study. The microbubbles were left at ambient room temperature for 30 min before being activated using a Vialmix (Lantheus Medical Imaging) for 45 s. Subsequently, the microbubbles were diluted with saline to a concentration of 1% (v/v) of mean mouse blood volume, which corresponds to 1 mL/kg. A volume of 100 µL of the diluted microbubble solution was injected into each animal immediately prior to sonication via a tail vein catheter, followed by a 150 µL saline (supplemented with 0.2% heparin) flush.

### Ultrasound Treatment

**Figure 1.**
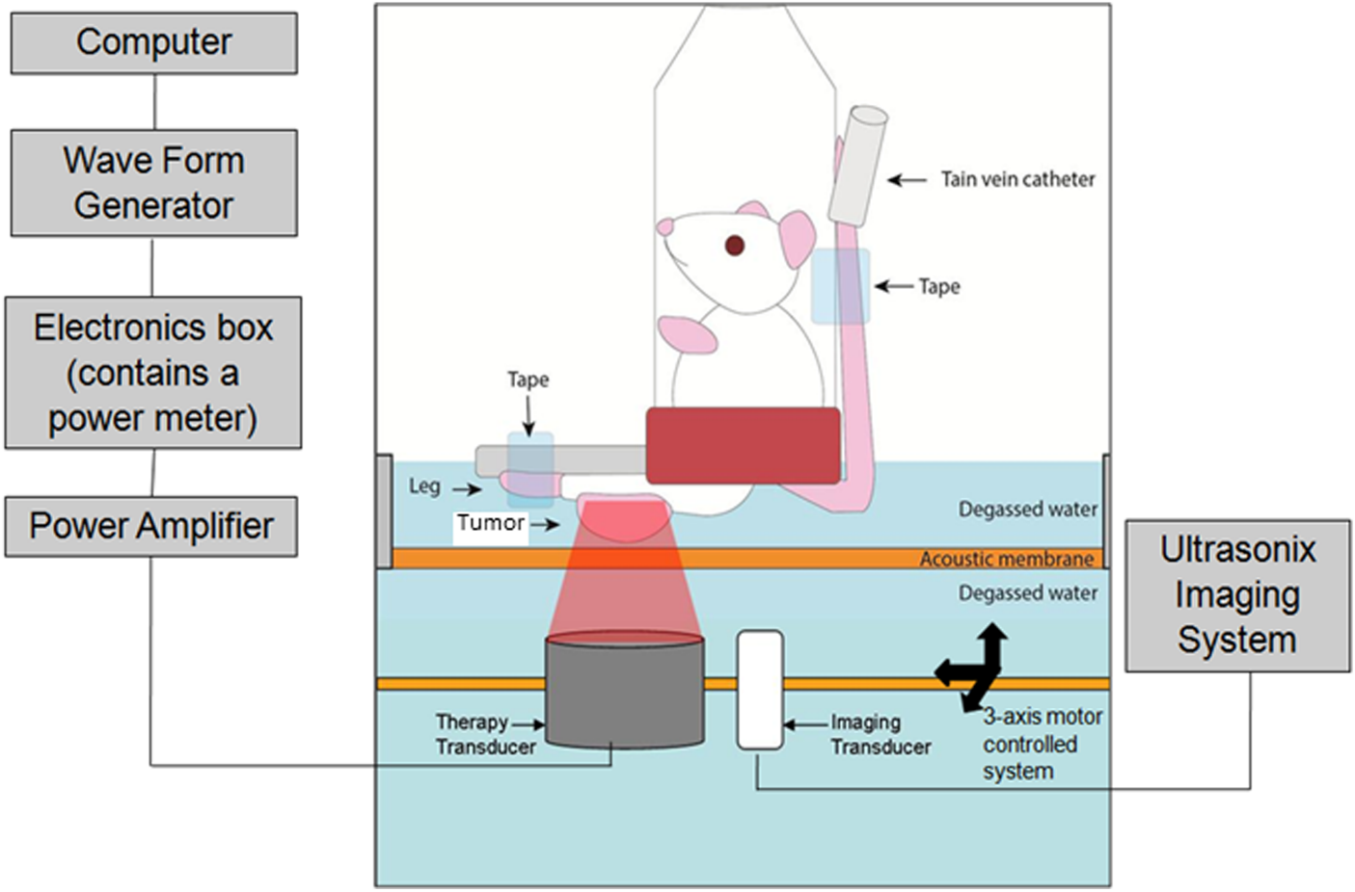
Schematic diagram of FUS treatment setup. Schematic diagram of the focused ultrasound (FUS) treatment system. The mouse is placed in a plastic tube and the leg is fixed to allow the tumor to face downwards. The focused ultrasound therapy transducer is guided by an imaging transducer.

An RK100 FUS therapy system (FUS Instruments, Toronto, Ontario, Canada) was used in this study. The device consists of a waveform generator, an electronics box containing a power meter, and an amplifier that is connected to a spherically focused piezoelectric therapy transducer. The therapy transducer has a 1.18-inch diameter, a 2.36-inch radius of curvature, a 488 kHz center frequency, and delivered pulses with 570 kPa peak negative pressure. The therapy transducer was positioned facing upwards next to a 10 MHz L14-5/8 imaging transducer connected to an Ultrasonix (BK Ultrasound, MA, USA) imaging system (Figure 1). The imaging transducer was used to locate the center of the tumor where a treatment target was selected. The therapy transducer was then electronically guided by a computer-controlled, 3-axis motorized, positioning system, such that the transducer focus was placed at the center of the selected treatment target.

Pulses, each lasting 32 µs, and with a 3 kHz pulse repetition frequency were sent in 50 ms tone bursts followed by a 1.95 s delay. This pulsing sequence was repeated for a total treatment time of 5-min. Within the 5-min treatment duration, a total of 150 bursts were sent, which resulted in a total insonation time of 750 ms and a 0.25% duty cycle. During treatment, the mouse was secured in an upright position with the tumor submerged in water. Once the therapy transducer was focused at the center of the treatment target, microbubbles were administered through the tail-vein catheter followed by a heparin-supplemented saline flush. Immediately upon microbubble injection, the tumors were exposed to ultrasound for 5 min.

### Radiation Therapy

Tumors were exposed to 160-kVp X-rays for a dose of 8 Gy at 200 cGy/min dose rate using a cabinet irradiator (Faxitron X-ray, IL, USA) immediately after the FUS  +  MB treatment. During irradiation, the animal's body was covered with a 3 mm-thick lead sheet, with the tumor exposed through a circular cut-out.

### Micro-Ultrasound Doppler Imaging

In this study, power Doppler imaging was used to detect blood flow in tumor vasculature pretreatment and at 24 h post-treatment. Data was acquired using a VEVO-770 system (VisualSonics, Toronto, Canada) with a VEVO RMV 710B transducer with a central frequency of 25 MHz. Three-dimensional (3D) power Doppler imaging was carried out with a step size of 0.2 mm, a wall filter of 2.5 mm∕s, a scan speed of 2 mm∕s, medium velocity, and a 20-dB gain setting. In-house software developed in MATLAB (Mathworks Inc, MA, USA) was used to analyze power Doppler data and calculate a vascularization index (VI). The VI is defined as the fraction of tumor volume that is occupied by the Doppler signal.

The animals were anesthetized with the ketamine and xylazine mixture during tumor imaging, and body temperature was maintained by resting the animal on a heating pad. The tumor bearing leg was stretched through an opening on the side of a weighing boat and secured with surgical tape, while deionized water was used as a coupling medium for ultrasound propagation. The water was heated to 37 °C to ensure normal blood flow.

### Histology Preparation

Twenty-four-hour after treatment administration, mice were sacrificed by cervical dislocation, and tumors were excised and fixed in 10% neutral-buffered formalin for 24 h at room temperature. The fixed tissue samples were then embedded in paraffin and sectioned into 5 µm slices for staining. Terminal deoxynucleotidyl transferase dUTP nick end labeling (TUNEL) was used to mark regions of apoptotic cell death by labeling fragmented DNA. Hematoxylin and eosin (H&E) staining was used to evaluate gross tumor destruction. The cluster of differentiation 31 (CD31) staining was used to assess tumor vascularization by marking endothelial cells lining the blood vessels within the tumor. In addition, Ki-67 labeling, which marks a nuclear protein present only in actively dividing cells,^24^ was used to identify the fraction of proliferating cells in tumors. Furthermore, carbonic anhydrase 9 (CA-9) labeling, a protein that is expressed in an acidic environment, which is associated with hypoxia,^25^ was used to identify regions of hypoxia within tumors under different treatment conditions. Finally, to investigate the mechanism of enhanced cell death, ceramide labeling was performed.

### Statistical Analysis

 Statistical significance was determined using Prism (GraphPad Software Inc., La Jolla, CA, USA) one-way analysis of variance followed by Šidák comparison test. A *P*-value of *P < .05, **P < .01, ***P < .001, ****P < .0001 was considered to be statistically significant. Each treatment condition was compared to the control (untreated) group. The statistical results for power Doppler and immunohistochemistry comparing each group are presented in supplementary data (S1–S6 Tables).

## Results

**Figure 2.**
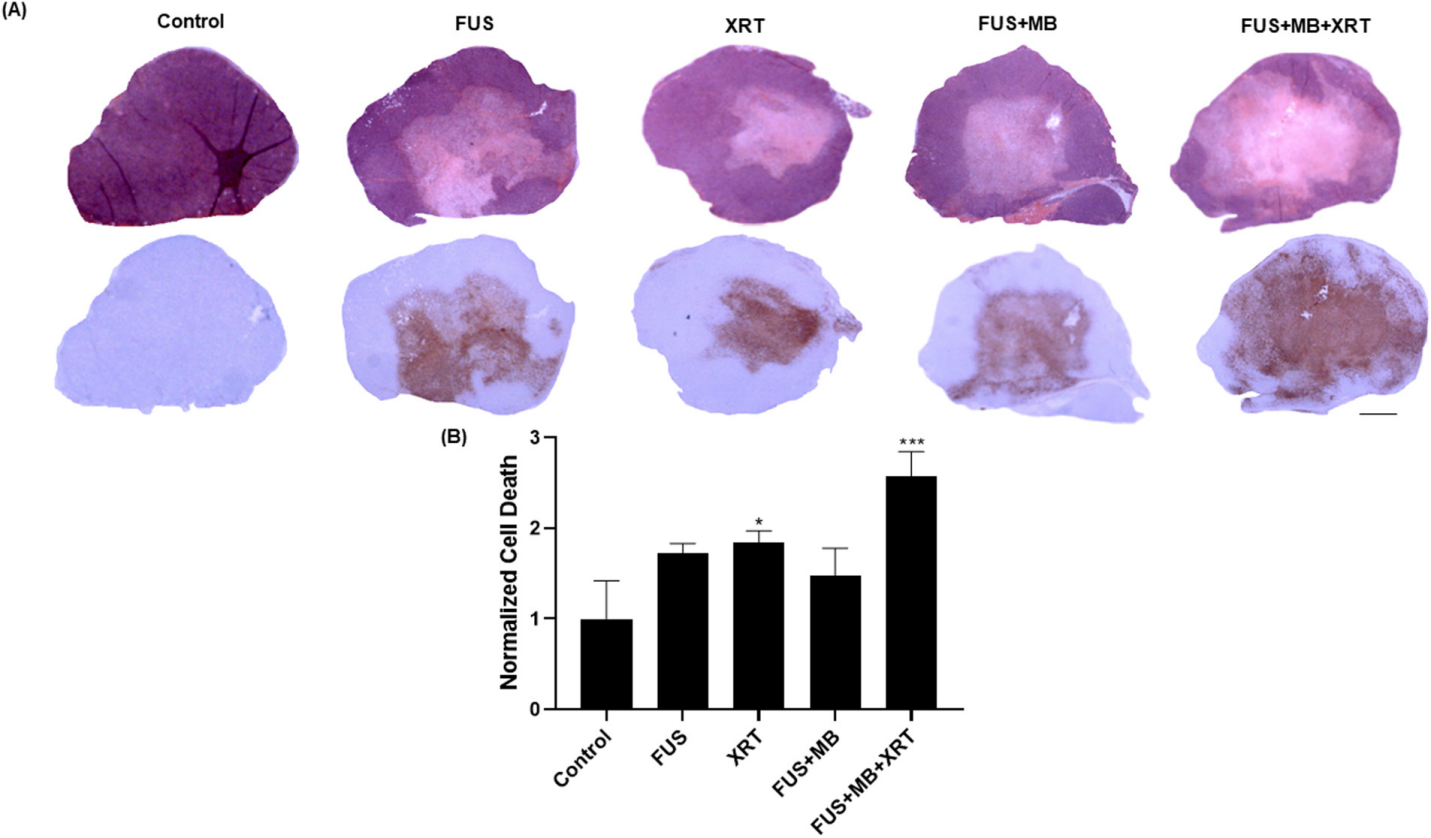
TUNEL and, H&E for cell death of MDA-MB-231 xenografts. (A) Low magnification light microscope images (obtained at 1X magnification) of MDA-MB-231 xenografts. The top row depicts H&E staining. The scale bar represents 2 mm. (B) Quantification of TUNEL stain representing cell death at 24 h after treatment. Error bars represent the standard error of the mean. *N*  =  5 animals per condition. Abbreviations: H&E, hematoxylin and eosin; TUNEL, terminal deoxynucleotidyl transferase dUTP nick end labeling.

In this study, the effect of combining localized ultrasound and microbubble treatment with XRT was assessed using power Doppler imaging and immunohistochemistry. A schematic of the experimental setup is depicted in Figure 1. Tumors treated with a combination of FUS-stimulated microbubbles (FUS  +  MB), or XRT demonstrated a significant increase in TUNEL staining compared to the untreated control, indicating an increase in apoptotic cell death 24 h after treatment administration. This effect was also observed in H&E sections (Figure 2A). The tumor area with positive TUNEL staining was quantified and is presented in Figure 2B. In comparison to control, tumors treated with a single 8 Gy doses of XRT alone or a combined treatment of (FUS  +  MB  +  XRT) demonstrated significant increase in cell death by 1.84 and 2.57 fold, respectively.

**Figure 3.**
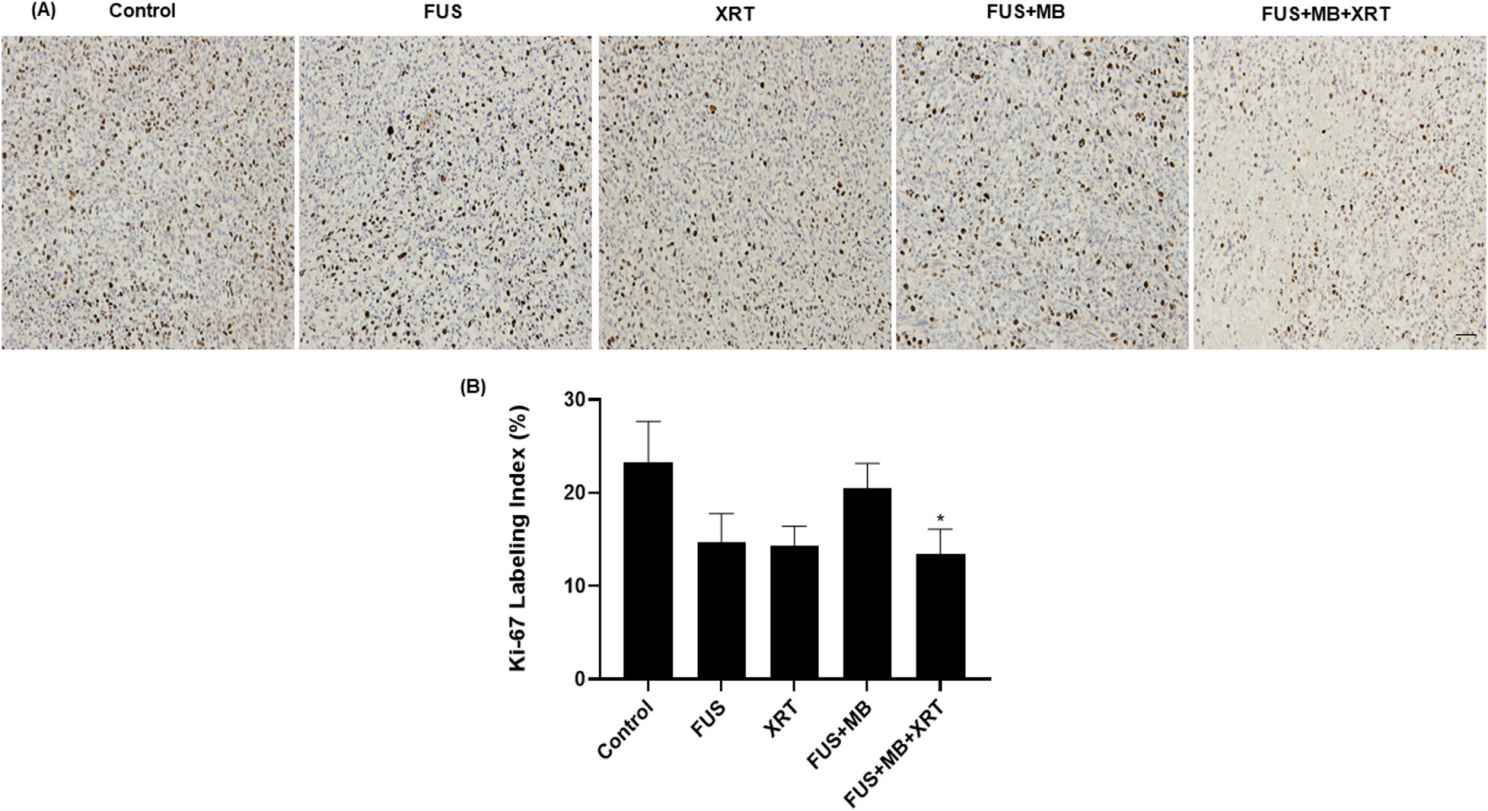
Ki-67 labeling of MDA-MB-231 tumor sections. (A) High magnification (acquired at 10× magnification) image of Ki-67-stained slides. The scale bar represents 50 µm. (B) Ki-67 quantification of immunohistochemical staining. Error bars represent the standard error of the mean. *N*  =  5 animals per condition.

In order to estimate the proliferation activity of tumor cells, Ki-67 immunolabeling was conducted (Figure 3). Tumors that received no treatment showed a Ki-67 labeling index of 23  ±  4% (mean  ±  SE), while tumors treated with FUS alone or a single dose of 8 Gy yielded a labeling index of 15  ±  3% and 14  ±  2%, respectively. Furthermore, tumors treated with FUS  +  MB yielded a Ki-67 labeling index of 21  ±  3% whereas those receiving the combined FUS  +  MB  +  XRT treatment demonstrated a significant decrease in Ki-67 labeling index to 13  ±  3% compared to control.

**Figure 4.**
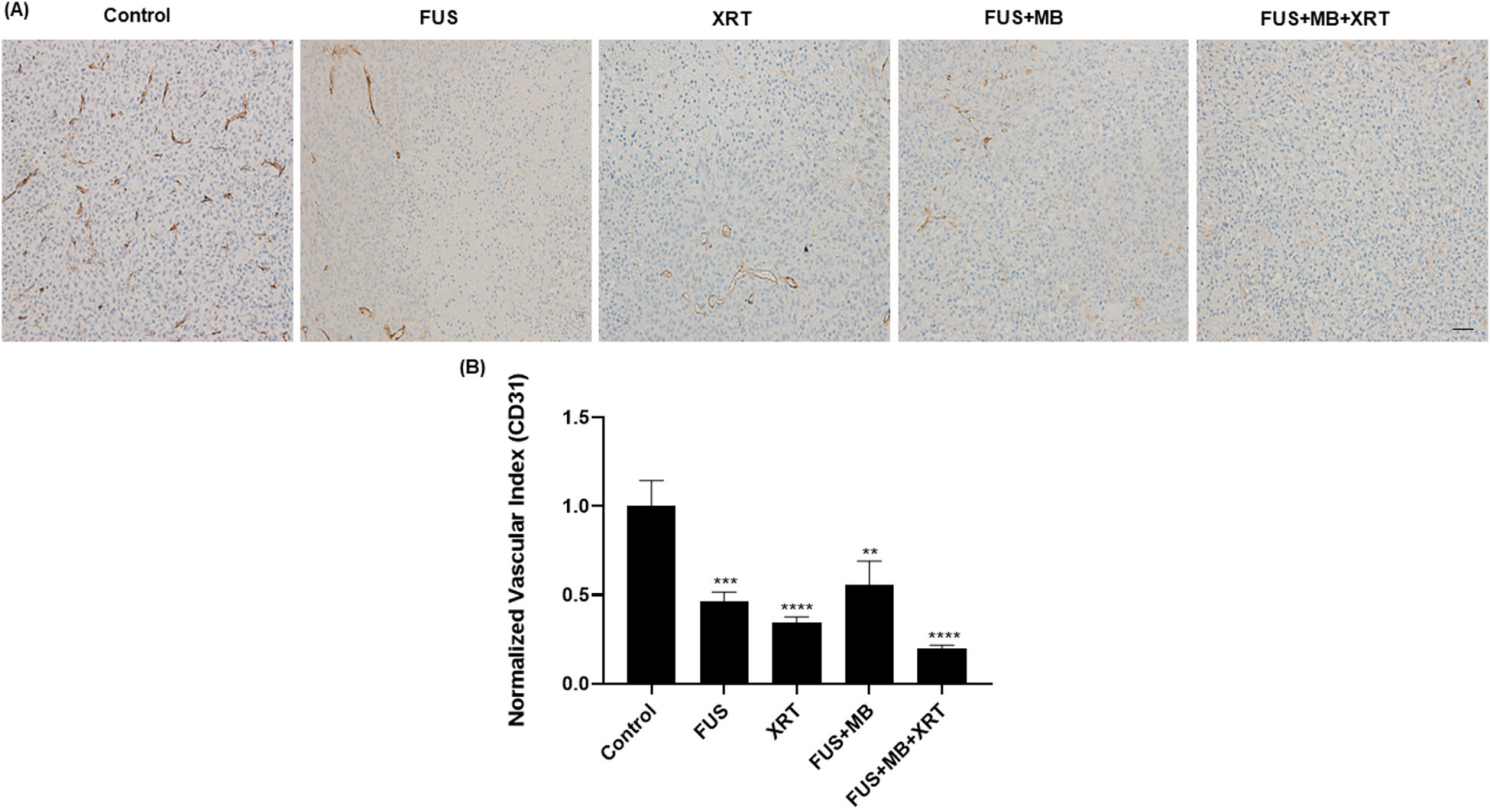
CD31 labeling of MDA-MB-231 xenograft tumors sections. (A) High magnification (acquired at 10×) images of a cluster of differentiation 31 (CD31)-stained slides. The scale bar represents 50 µm (B) Normalized vascular index obtained from CD31 analysis following different treatments. Error bars represent the standard error of the mean. *N*  =  5 animals per condition.

CD-31 immunohistochemical analysis was used to mark endothelial cells and the degree of tumor vascularization. Intact appearing endothelial cells were counted in 5 randomly selected regions of interest per tumor section. The results demonstrated a decrease in intact, normal-appearing vascularization in treated groups compared to the untreated control group (Figure 4). The normalized vascular index decreased from a value of 1.00  ±  0.14 in the untreated control group to 0.5  ±  0.1 (*P*  =  .0006), 0.34  ±  0.03 (*P* < .0001), and 0.6  ±  0.1 (*P*  =  .003) in the FUS only, XRT only, and FUS  +  MB groups, respectively. The vascular index in the samples that received a combination of FUS  +  MB  +  XRT significantly decreased to a value of 0.2  ±  0.02 (*P* < .0001).

**Figure 5.**
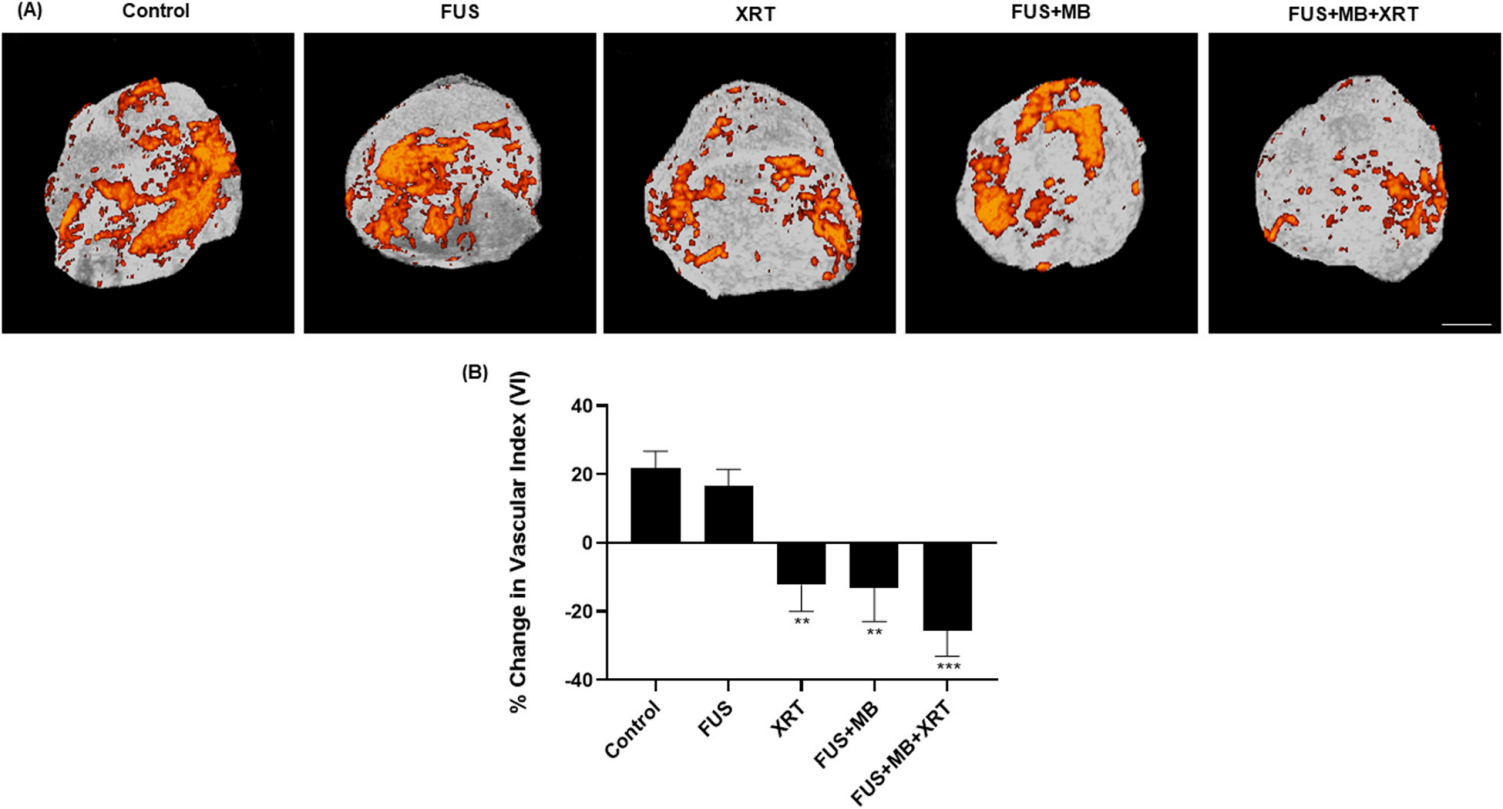
Twenty-four-hour response monitoring of tumor blood flow. (A) Representative three-dimensional (3D) power Doppler images of treatment effects. The magnification bar represents 1mm. (B) Quantification of blood flow indicating vascular index change before and after treatment. Error bars represent the standard error of the mean. *N*  =  5 animals per condition.

The relative change in the power Doppler vascular index before and at 24 h after treatment was assessed in this study. The results demonstrated a reduction in the vascular index in treated samples compared to the untreated control. The vascular index in the untreated control was 22  ±  5%. The FUS-only group demonstrated a vascular index of 17  ±  5%. Treatment with 8 Gy single-dose XRT resulted in a −12  ±  8% decrease in power Doppler vascular index (*P*  =  .0029) while a FUS  +  MB treatment alone yielded a −13  ±  10% (P = .0023) decrease in vascular index. The combination of FUS  +  MB  +  XRT resulted in a −25  ±  8% decrease at 24 h post-treatment (*P*  =  .0001) (Figure 5).

**Figure 6.**
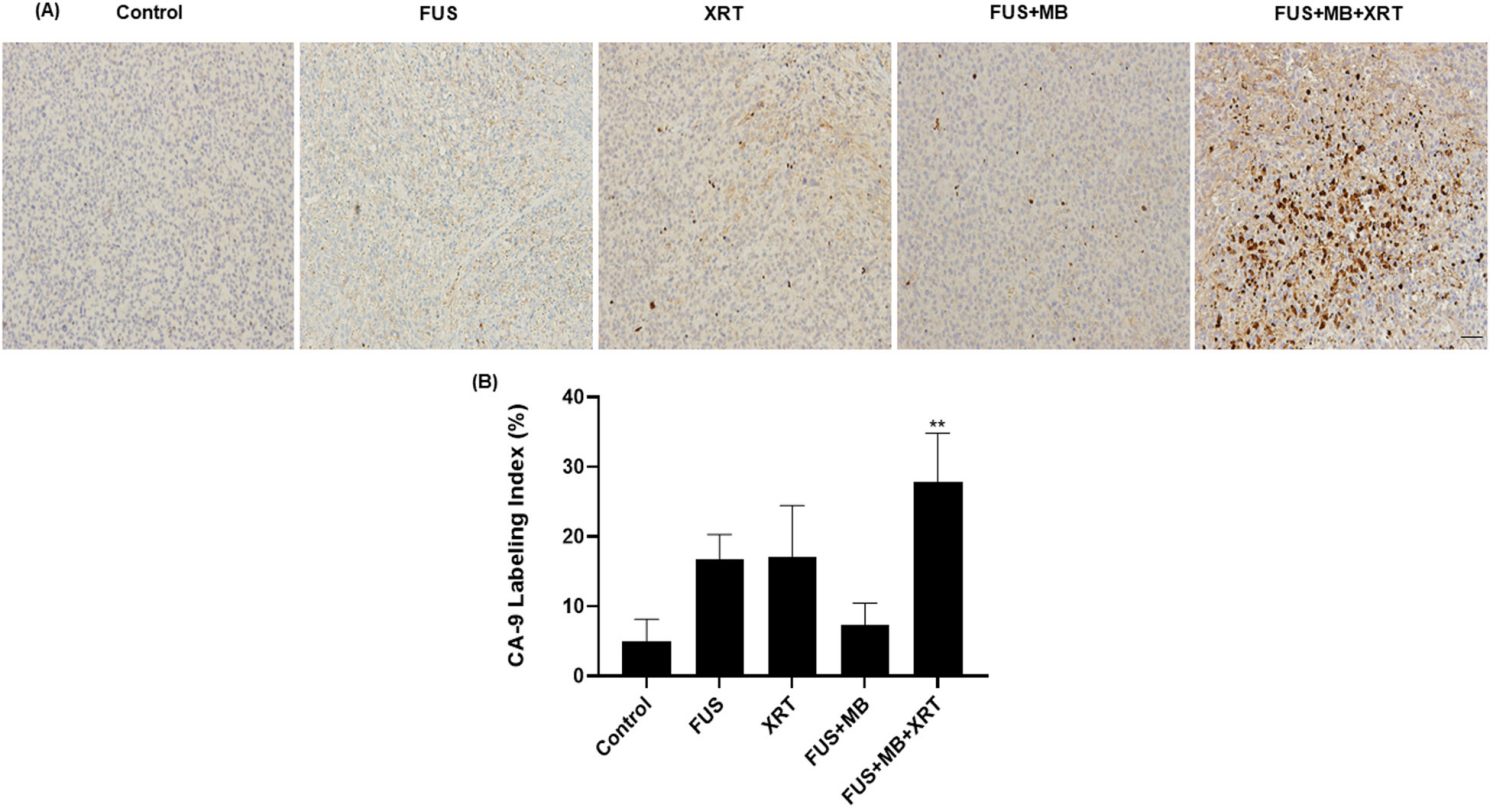
(A) Images of carbonic anhydrase (CA-9) labeling and quantification of xenograft MDA-MB-231 tumors treated with various conditions. High magnification (acquired at 10×) images of CA-9-stained slides are shown. The scale bar represents 50 μm (B) Quantitative analysis of CA-9 labeling index. Error bars represent the standard error of the mean. *N* = 5 animals per condition.

In order to investigate regions of hypoxia in the tumor section, CA-9 labeling was performed. The group that were exposed to FUS only, XRT only and FUS  +  MB demonstrated a CA-9 labeling index of 17  ±  4%, 17  ±  7% and 7  ±  3.2%, respectively (Figure 6). The highest level of hypoxic areas (28  ±  7%) occurred in tumors treated with FUS  +  MB  +  XRT. This was 5.6-fold higher than the percentage of hypoxia resulting from control group (5  ±  3%).

**Figure 7.**
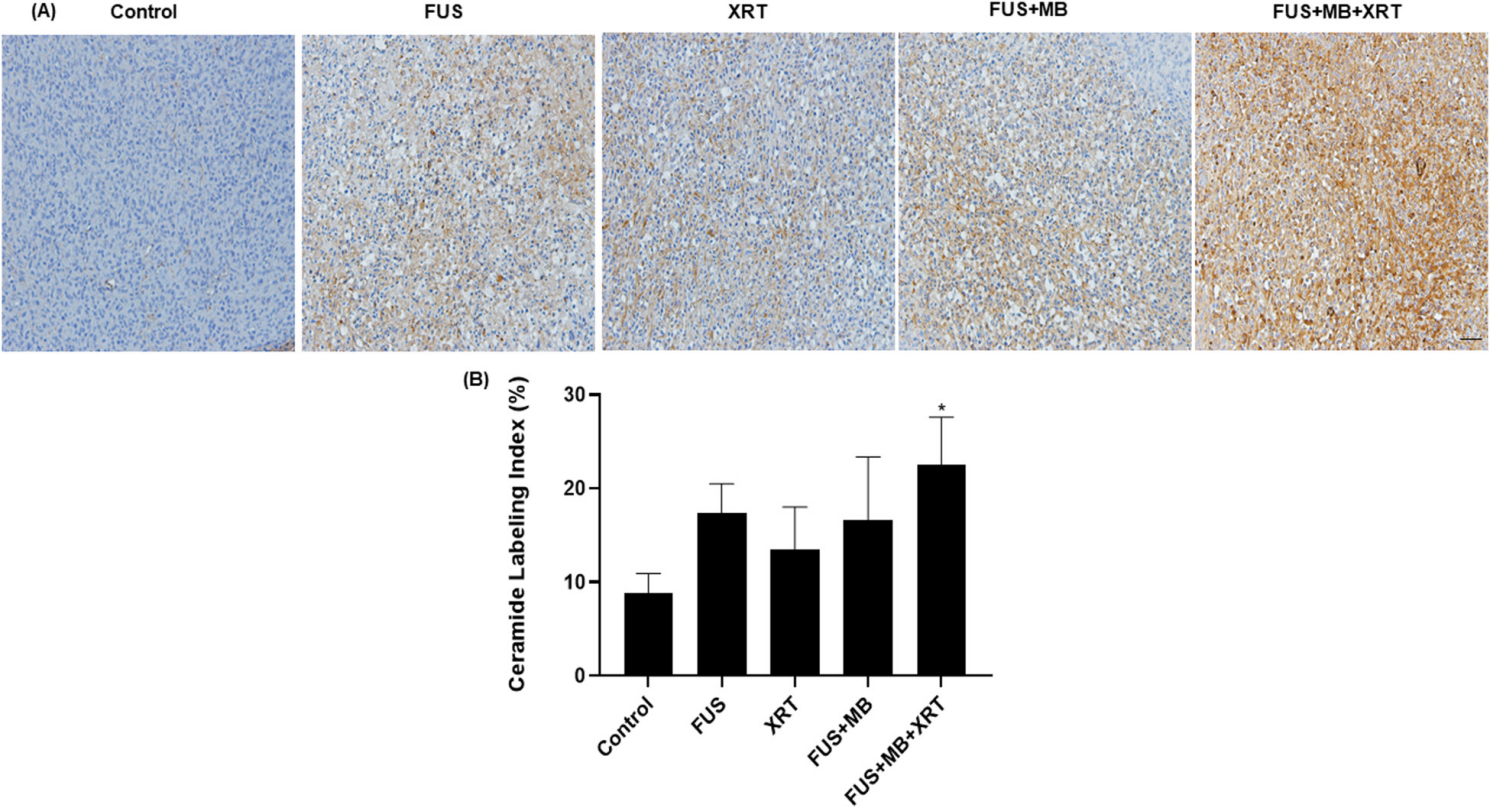
Ceramide-stained sections from MDA-MB-231 xenograft tumors. (A) High magnification (acquired at 10× magnification) images of ceramide-stained slides. The scale bar represents 50 µm (B) Quantified ceramide staining. Error bars represent the standard error of the mean. *N*  =  5 animals per condition.

Ceramide labeling was conducted in order to evaluate the production of ceramide in tumor sections (Figure 7). Compared to the control group, tumors treated with FUS  +  MB  +  XRT exhibited a significant increase in ceramide labeling index by 2.5-fold. The ceramide labeling indices of tumors exposed to FUS alone, XRT alone, or FUS  +  MB remained at 17  ±  3%, 14  ±  5% or 17  ±  7%, respectively.

## Discussion

This study investigated the effects of using acoustically driven microbubbles in combination with XRT and tested the hypothesis that a combination of FUS-stimulated microbubbles and XRT treatment can enhance the therapeutic outcomes in breast cancer xenografts in vivo. It is believed that the main mechanism of FUS-stimulated microbubble-enhanced XRT is the mechanical disruption of tumor microvasculature through acoustic cavitation.^12^ The results obtained from this study demonstrated that FUS-stimulated microbubbles enhanced treatment outcomes when combined with XRT. In this study, tumor response was evaluated at 24 h. Based on our previous findings, 24-h time duration demonstrated maximal tumor response, however, after this time point the response seems to be minimum. Previous studies have indicated that MDA-MB-231 xenograft upon exposure to USMB and chemotherapy or radiotherapy caused the highest amount of tumor cell death and vascular damage at 24 h, indicating greater tumor response.^26,27^ Therefore, in our present study, we anticipated that incorporating 24 h to monitor tumor response would ensure maximum effectiveness of the treatment.

In the study here, treatment with FUS-stimulated microbubbles combined with radiation demonstrated a significantly increased tumor cell death. These results were consistent with previous studies conducted with prostate, bladder, and breast cancer xenografts.^4-6,19^ The observed increase in cell death (Figure 2) was also accompanied by a decrease in the proliferative fraction of tumor cells demonstrated by a decrease in Ki-67 labeling in the treated tumors (Figure 3). This was expected based on previous work and consistent with TUNEL results, where the combined treatment resulted in the highest cell death index and correspondingly had the lowest proliferative fraction. Comparable results were reported in previous studies on prostate cancer xenografts that were treated with similar treatment conditions and assessed 24 h after treatment.^4^ This decrease in Ki-67 labeling also supports the observation made by Lai et al,^19^ in breast cancer xenografts where tumors that were treated with a combination of USMB and radiation had slower tumor growth rates compared to untreated tumors in long term studies.^19^

Several studies have demonstrated the feasibility of using high-frequency power Doppler imaging to assess tumor vascular response.^18,28^ A reduction in power Doppler signal indicates a reduction in blood flow within the tumor.^18^ In the work here, a significant decrease in blood flow compared to the untreated control was observed in tumors that were treated with FUS and microbubbles only and radiation only (Figure 5). However, the most significant decrease was observed in the tumors that received a combination of the 2 treatments. These results were consistent with previous studies conducted on bladder cancer xenografts. The decrease in blood flow was consistent with CD31 immunohistochemical analysis, where a significant reduction in tumor vascularization was observed (Figure 4). The decreases in tumor vascularization and blood flow observed here were accompanied by an increase in CA-9 labeling compared to the untreated control (Figure 6). The CA-9 protein is over-expressed in hypoxic cells. The increased hypoxia could be a direct result of vascular disruption and reduced perfusion in tumor vasculature.^29^

One of the hypothesized mechanisms of FUS-stimulated microbubble-enhanced XRT is that, when injected intravenously and stimulated by ultrasound, microbubbles can exert shear stress on neighboring endothelial cells lining blood vessels causing membrane damage. This disruption of tumor vascular endothelial cells can lead to the activation of a ceramide-mediated cell signaling pathway that triggers apoptosis, hence enhancing tumor cell killing in response to XRT.^30^ To verify this mechanism, ceramide labeling was conducted in this study. The results indicated an increase in ceramide levels in treated samples compared to the untreated control (Figure 7). However, the increase in ceramide production was only statistically significant in the group that received the combined treatment. This is consistent with the general results obtained from TUNEL staining linked to cell death and suggests that the increased ceramide levels are a potential cause of increased cell death. It has been demonstrated by previous studies that ceramide production can increase significantly in cancer cells as well as in endothelial cells in response to XRT and USMB exposure.^20,30^ Combining USMB treatment with XRT can result in vascular disruption by damaging endothelial cells lining blood vessels and decreasing blood flow to the tumor. This, in turn, can result in decreased tumor oxygenation and tumor cell proliferation, and increased cell death. In addition, endothelial cell damage induced by both USMB exposure and radiation increases ceramide production and enhances the ceramide-mediated apoptosis pathway, leading to further increases in cell death. The involvement of ceramide in vascular disruption subsequently accompanied by cell death has been extensively investigated.^31^ A study conducted by Al-Mahrouki et al^4^ explored the signaling pathway involved in response to ceramide activation/production causing substantial damage to vasculature followed by USMBs and XRT. In other work, a gene responsible for membrane biogenesis and repair involved in the transfer of galactose to ceramide, UDP glycosyltransferase 8 (UGT8) was experimentally upregulated or downregulated in prostate cancer xenografts. Results demonstrated that xenografts with down-regulated UGT8 gene exhibited a higher accumulation of ceramide followed by significant cell death leading to a reduction in blood flow and oxygen saturation level compared to control (untreated). On contrary, the reverse phenomenon was observed in xenografts with upregulated UGT8 levels.^31^

Another explored mechanism of radiation-induced cancer cell death is by overcoming tumor hypoxia. The treatment outcome with radiotherapy is known to be greatly influenced by hypoxia.^32–34^ Preclinical data suggests radiation activates and upregulates hypoxia-inducible factor 1 (HIF-1) levels, promoting radioresistance. The activation and accumulation of HIF-1 is known to be caused due to reoxygenation after irradiation.^35^ Several attempts have been made to restore the oxygen content in the tumor cells, one of which includes delivery of microbubbles carrying oxygen. An increase of 20 mmHg oxygen content in the breast tumor model has been documented using the delivery of ultrasound-triggered oxygen-filled microbubbles. Defeating hypoxia using this technique prior to radiotherapy demonstrated greater radiosensitivity.^36,37^ It is still unclear if oxygen carrying microbubbles have any influence in radiation-induced ceramide production. It would be interesting to see if microbubble carrying oxygen improves the response to XRT is associated with ceramide production.

The results obtained from the current study are consistent with the findings of previous studies done using more simplistic ultrasound therapy on breast, prostate, and bladder cancer.^4-6,19^ However, the current study improves the spatial specificity of the treatment by using image guidance and FUS therapy, which allows for concentrating ultrasound energy in a small focal area and improves the penetration of the ultrasound beam. This demonstrates initial workings toward a framework to treat deeper targets. It has to be pointed out that our previous studies^5,19^ have shown synergistic effects following USMB and radiation in an in vivo xenograft model however, in this study no synergistic effect of FUS-stimulated microbubbles and radiation was detected. The rationale for not observing synergy here could be treatment dependent. In those studies, different xenograft types and different concentrations of microbubbles were used. However, this needs to be validated in future work. Thus, overall the study here demonstrates enhanced tumor response with combined treatment of FUS-stimulated microbubble and XRT. Even though the outcomes hold a promising future for clinical settings, the limitations of this study cannot be overlooked. Several limitations to this study are included in the following points. In the present work, the impact of FUS+MB and XRT on tumor blood vessels was detected using CD31 immunohistochemistry and power Doppler ultrasound. However, both these techniques are unable to differentiate between perfused vessels from nonperfused ones. It is therefore essential to consider perfusion assays or perfusion imaging techniques to better understand the tumor vascular architecture in a more precise manner. Another limitation of this study is the usage of the xenograft model, which does not completely recapitulate human tumor biology. Instead, using more clinically relevant orthotopic models, specifically patient-derived cell xenografts might help mimic human tumor vasculature closely and can help predict clinical outcomes more accurately. Another limitation of the current work is the assessment of treatment response acutely (at 24 h). Even though enhanced tumor response with increase tumor cell death and vascular damage following treatments have been validated in our study, this does not directly translate into clinical settings. A longitudinal study including multiple treatment regimens to examine the treatment effects and its potential risk factors should be included in future work. In addition, monitoring tumor growth over a longitudinal period might help in treatment response prediction and switch treatments if required at its earliest.

## Conclusion

This study demonstrated that combining a single dose of XRT with USMBs improved treatment effects in a breast cancer xenograft model. The results indicate the possibility that, lower doses of radiation when combined with USMBs, may have the same effect as higher doses of radiation in breast tumors. Targeted stimulation of microbubbles at the tumor site can be achieved using FUS and improved precision of treatment targeting can be enhanced using image guidance. The research presented in this paper is the foundation for future research that examines the use of image-guided FUS and microbubble treatment in combination with XRT in larger tumors grown in more complex animals.

## Supplemental Material

sj-docx-1-tct-10.1177_15330338221132925 - Supplemental material for Focused Ultrasound Stimulation of Microbubbles in Combination With Radiotherapy for Acute Damage of Breast Cancer Xenograft Model Click here for additional data file. Supplemental material, sj-docx-1-tct-10.1177_15330338221132925 for Focused Ultrasound Stimulation of Microbubbles in Combination With Radiotherapy for Acute Damage of Breast Cancer Xenograft Model by Deepa Sharma, PhD, Farah Hussein, MSc, Niki Law, BSc MRT(t), Golnaz Farhat, PhD, Christine Tarapacki, MSc, Lakshmanan Sannachi, PhD, Anoja Giles, BSc, and Gregory J. Czarnota, MD, PhD in Technology in Cancer Research & Treatment
