# Supplementary material for: Focused Ultrasound Stimulation of Microbubbles in Combination With Radiotherapy for Acute Damage of Breast Cancer Xenograft Model
Source: Technol Cancer Res Treat. 2022 Nov 22;21:15330338221132925. doi: 10.1177/15330338221132925 (PMC9706051; doi:10.1177/15330338221132925)
Supplement: sj-docx-1-tct-10.1177_15330338221132925 - Supplemental material for Focused Ultrasound Stimulation of Microbubbles in Combination With Radiotherapy for Acute Damage of Breast Cancer Xenograft Model [file sj-docx-1-tct-10.1177_15330338221132925.docx]

**S1 Table. Statistical analysis of cell death. Results of statistical analysis performed on the “Quantification of Cell death” using one-way ANOVA followed by Šidák comparison test.**

| **Comparison** | **P-value** | **Significance** |
| --- | --- | --- |
| Control vs FUS | 0.0771 | ns |
| Control vs XRT | 0.0409 | * |
| Control vs FUS+MB | 0.2353 | ns |
| Control vs FUS+MB+XRT | 0.0006 | *** |
|  |  |  |
| FUS vs FUS+MB | 0.5294 | ns |
| FUS vs FUS+MB+XRT | 0.0387 | * |
|  |  |  |
| XRT vs FUS+MB+XRT | 0.0732 | ns |
|  |  |  |
| FUS+MB vs FUS+MB+XRT | 0.0098 | ** |

**S2 Table. Statistical analysis of Ki-67. Results of statistical analysis performed on the “Quantification of Ki-67” using one-way ANOVA followed by Šidák comparison test.**

| **Comparison** | **P-value** | **Significance** |
| --- | --- | --- |
| Control vs FUS | 0.0679 | ns |
| Control vs XRT | 0.0570 | ns |
| Control vs FUS+MB | 0.5466 | ns |
| Control vs FUS+MB+XRT | 0.0392 | * |
|  |  |  |
| FUS vs FUS+MB | 0.2228 | ns |
| FUS vs FUS+MB+XRT | 0.7952 | ns |
|  |  |  |
| XRT vs FUS+MB+XRT | 0.8609 | ns |
|  |  |  |
| FUS+MB vs FUS+MB+XRT | 0.1436 | ns |

**S3 Table. Statistical analysis of CD31. Results of statistical analysis performed on the “Quantification of CD31” using one-way ANOVA followed by Šidák comparison test.**

| **Comparison** | **P-value** | **Significance** |
| --- | --- | --- |
| Control vs FUS | 0.0006 | *** |
| Control vs XRT | <0.0001 | **** |
| Control vs FUS+MB | 0.0033 | ** |
| Control vs FUS+MB+XRT | <0.0001 | **** |
|  |  |  |
| FUS vs FUS+MB | 0.4756 | ns |
| FUS vs FUS+MB+XRT | 0.0593 | ns |
|  |  |  |
| XRT vs FUS+MB+XRT | 0.2834 | ns |
|  |  |  |
| FUS+MB vs FUS+MB+XRT | 0.0130 | * |

**S4 Table. Statistical analysis of power Doppler. Results of statistical analysis performed on the “Quantification of power Doppler” using one-way ANOVA followed by Šidák comparison test.**

| **Comparison** | **P-value** | **Significance** |
| --- | --- | --- |
| Control vs FUS | 0.6128 | ns |
| Control vs XRT | 0.0029 | ** |
| Control vs FUS+MB | 0.0023 | ** |
| Control vs FUS+MB+XRT | 0.0001 | *** |
|  |  |  |
| FUS vs FUS+MB | 0.0076 | ** |
| FUS vs FUS+MB+XRT | 0.0005 | *** |
|  |  |  |
| XRT vs FUS+MB+XRT | 0.2056 | ns |
|  |  |  |
| FUS+MB vs FUS+MB+XRT | 0.2382 | ns |

**S5 Table. Statistical analysis of CA-9. Results of statistical analysis performed on the “Quantification of CA-9” using one-way ANOVA followed by Šidák comparison test.**

| **Comparison** | **P-value** | **Significance** |
| --- | --- | --- |
| Control vs FUS | 0.1311 | ns |
| Control vs XRT | 0.1214 | ns |
| Control vs FUS+MB | 0.7601 | ns |
| Control vs FUS+MB+XRT | 0.0061 | ** |
|  |  |  |
| FUS vs FUS+MB | 0.2204 | ns |
| FUS vs FUS+MB+XRT | 0.1516 | ns |
|  |  |  |
| XRT vs FUS+MB+XRT | 0.1631 | ns |
|  |  |  |
| FUS+MB vs FUS+MB+XRT | 0.0122 | * |

**S6 Table. Statistical analysis of Ceramide. Results of statistical analysis performed on the “Quantification of Ceramide” using one-way ANOVA followed by Šidák comparison test.**

| **Comparison** | **P-value** | **Significance** |
| --- | --- | --- |
| Control vs FUS | 0.1772 | ns |
| Control vs XRT | 0.4541 | ns |
| Control vs FUS+MB | 0.2172 | ns |
| Control vs FUS+MB+XRT | 0.0368 | * |
|  |  |  |
| FUS vs FUS+MB | 0.9065 | ns |
| FUS vs FUS+MB+XRT | 0.4341 | ns |
|  |  |  |
| XRT vs FUS+MB+XRT | 0.1749 | ns |
|  |  |  |
| FUS+MB vs FUS+MB+XRT | 0.3699 | ns |
